# A Quantum Chemistry Approach to Linear Vibro-Polaritonic Infrared Spectra with Perturbative Electron–Photon Correlation

**DOI:** 10.1021/acs.jpclett.4c00105

**Published:** 2024-02-21

**Authors:** Eric W. Fischer, Jan A. Syska, Peter Saalfrank

**Affiliations:** † Institut für Chemie, Humboldt-Universität zu Berlin, Brook-Taylor-Straße 2, D-12489 Berlin, Germany; ‡ Institut für Chemie, Universität Potsdam, Karl-Liebknecht-Straße 24-25, D-14476 Potsdam-Golm, Germany; § Institut für Physik und Astronomie, Universität Potsdam, Karl-Liebknecht-Straße 24-25, D-14476 Potsdam-Golm, Germany

## Abstract

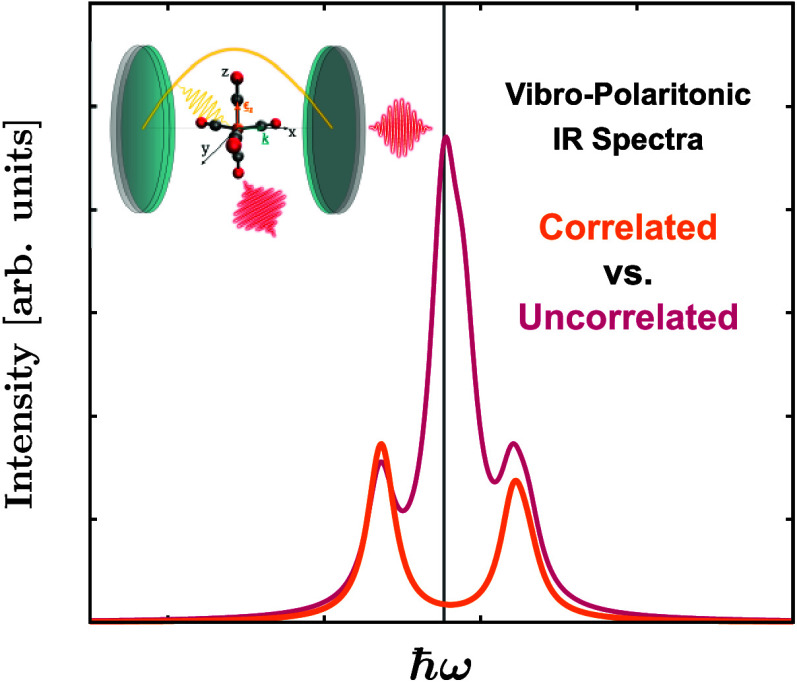

In the vibrational strong coupling (VSC) regime, molecular vibrations and resonant low-frequency cavity modes form light–matter hybrid states, vibrational polaritons, with characteristic infrared (IR) spectroscopic signatures. Here, we introduce a molecular quantum chemistry-based computational scheme for linear IR spectra of vibrational polaritons in polyatomic molecules, which perturbatively accounts for nonresonant electron–photon interactions under VSC. Specifically, we formulate a cavity Born–Oppenheimer perturbation theory (CBO-PT) linear response approach, which provides an approximate but systematic description of such electron–photon correlation effects in VSC scenarios while relying on molecular *ab initio* quantum chemistry methods. We identify relevant electron–photon correlation effects at the second order of CBO-PT, which manifest as static polarizability-dependent Hessian corrections and an emerging polarizability-dependent cavity intensity component providing access to transmission spectra commonly measured in vibro-polaritonic chemistry. Illustratively, we address electron–photon correlation effects perturbatively in IR spectra of CO_2_ and Fe(CO)_5_ vibro-polaritonic models in sound agreement with nonperturbative CBO linear response theory.

Vibrational polaritons are light–matter hybrid states formed when molecular vibrational modes strongly interact with quantized modes of optical low-frequency cavities^1^ underlying the emerging field of vibro-polaritonic chemistry.^2,3^ Experimentally, vibrational polaritons exhibit characteristic spectroscopic signatures, specifically transitions to upper and lower vibro-polaritonic states, which have been probed by both linear^4−9^ and nonlinear^10^ infrared (IR) spectroscopic techniques.

Computationally, linear vibro-polaritonic IR spectra are commonly obtained from linear response theory, where two distinct routes exist: (1) linear response approaches based on effective ground state Pauli–Fierz Hamiltonians, which are fully characterized by purely molecular properties, e.g., the molecular ground state potential energy surface (PES) and dipole moment, that can be easily accessed by means of standard quantum chemistry methods,^11−14^ and (2) a linear response approach formulated in the cavity Born–Oppenheimer (CBO) approximation,^15−18^ which relies on an extended electronic structure problem accounting for electron–photon correlation due to nonresonant interactions between electrons and low-frequency cavity modes.^18^ The CBO formulation is more complete but also more involved because generalized cavity PES and dipole moments depend on molecular and cavity coordinates. Both approaches rely on a linear response formulation in double-harmonic approximation, i.e., harmonic molecular and cavity modes in combination with a linearized ground state dipole moment. This is particularly beneficial for treating the vibrations and spectra of large molecules or molecular ensembles quantum mechanically.

Recently, we showed that effective ground state and CBO formulations differ in their description of electron–photon correlation.^19^ Specifically, effective ground state models can be understood from the perspective of a *crude* CBO approximation relying on the adiabatic electronic ground state, which completely neglects electron–photon correlation in the VSC regime.^19^ We proposed a perturbative connection between crude CBO and correlated CBO formulations, denoted CBO perturbation theory (CBO-PT), motivated by distinct excitation energy scales of electronic and low-frequency cavity mode subsystems.^19^ CBO-PT solves the extended CBO electronic structure problem perturbatively and allows for systematic electron–photon correlation corrections of the crude CBO approach.^19^ We note, CBO-PT is conceptually similar to other perturbative approaches,^20,21^ which however constitute a further approximation^19,20^ or do not directly connect to the CBO formulation.^21^

In this work, we introduce an approximation to the nonperturbative CBO linear response framework of Bonini and Flick^15^ by combining CBO-PT with linear response theory. This approach allows us to systematically correct vibro-polaritonic IR spectra for electron–photon correlation effects while fully relying on molecular properties that can be accessed by *ab initio* quantum chemistry methods. We derive explicit expressions for electron–photon correlation-corrected vibro-polaritonic Hessian matrix elements and IR intensities, which are both shown to be determined by the molecule’s static polarizability, whose possible relevance has been noted only recently.^21−23^ We illustratively apply the CBO-PT linear response approach to vibro-polaritonic model systems of CO_2_ and Fe(CO)_5_ (cf. Figure 1) in line with ref (15) and discuss electron–photon correlation effects in connection to experimentally relevant transmission spectra.^4,7^

**Figure 1:**
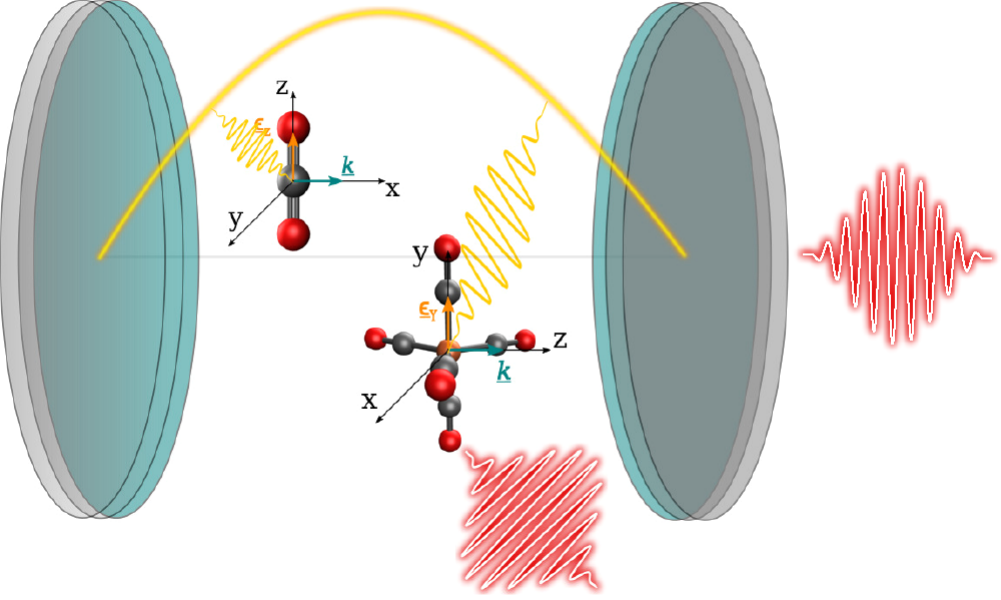
Sketch of CO_2_ and Fe(CO)_5_ molecules strongly coupled (yellow wavy lines) to the low-frequency cavity mode (yellow) with a cavity IR response (right red pulse) and a molecular IR response (bottom red pulse).

We first recapitulate the main aspects of CBO linear response theory,^15^ which fully accounts for electron–photon correlation in vibro-polaritonic IR spectra. We consider a molecular system with *N*_n_ nuclei under a VSC with 2*N*_c_ quantized transverse field modes of an IR cavity. We furthermore assume the CBO approximation to be valid; i.e., non-adiabatic coupling to the excited state manifold is negligible.^18,19^ The CBO electronic ground state problem is described by an electron–photon time-independent Schrödinger equation (TISE)^17,18^
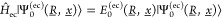
1with the adiabatic electron–photon ground state, |Ψ_0_^(ec)^⟩, and ground state cavity potential energy surface (cPES), *E*_0_^(ec)^, which parametrically depend on both nuclear, , and cavity displacement coordinates, . The electron–photon Hamiltonian reads^18^
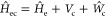
2with electronic Hamiltonian  composed of the electronic kinetic energy, , and the molecular Coulomb potential, *V*_coul_. In addition,  is the harmonic cavity potential characterized by harmonic frequencies, ω_*k*_, and displacement coordinates, *x*_*λk*_, with polarization λ and mode index *k*. The third term on the right-hand side of eq 2 constitutes the light–matter interaction potential
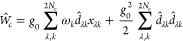
3with polarization-projected molecular dipole operator , cavity polarization vector , and light–matter interaction constant , which is determined by the cavity volume *V*_cav_ and the permittivity ε_0_, and relates to a mode specific equivalent,  (cf. section S1 of the Supporting Information). The first term in eq 3 is the bare light–matter interaction (*g*_0_), and the second term (*g*_0_^2^) resembles the dipole self-energy (DSE).^24^ couples the electronic subsystem (in addition to the nuclear one) to low-frequency cavity modes and contains a DSE-induced electron–electron interaction, which is independent of interparticle distance.^15,18,19^

In nonperturbative CBO linear response theory, the ground state cPES in eq 1 is harmonically approximated^15^
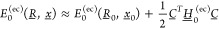
4around a minimum configuration, , with coordinate vector , which collects *N*_vib_ mass-weighted Cartesian displacement coordinates, , with atomic mass *M*_*i*_ and 2*N*_c_ cavity displacement coordinates, Δ*x*_*λk*_ = *x*_*λk*_ – *x*_*λk*_^0^. Vectors  and  collect all reference values, *R*_*i*,0_ and *x*_*λk*_^0^, respectively. The vibro-polaritonic Hessian, , contains second-order derivatives of the ground state cPES with respect to both mass-weighted molecular Cartesian and cavity displacement coordinates, and specifies a matrix eigenvalue problem^15^
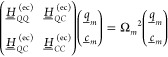
5where  is written as a 2 × 2-block matrix with molecular (*QQ*), cavity (*CC*), and light-matter interaction blocks (*QC*). Eigenvectors  resemble vibro-polaritonic normal modes with molecular, , and cavity, , components characterized by corresponding harmonic vibro-polaritonic frequencies, Ω_*m*_. The eigensystem of eq 5 provides access to linear vibro-polaritonic IR spectra
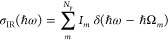
6for *N*_p_ = *N*_vib_ + 2*N*_c_ vibro-polaritonic normal modes. IR intensities, *I*_*m*_ = *∑*_κ_|*Z*_*mκ*_|^2^, are determined by Cartesian components of mode effective charges
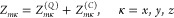
7with molecular and cavity mode contributions^15^
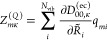
8
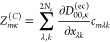
9where  and with *q*_*mi*_ and *c*_*mλk*_ being elements of  and , respectively, in eq 5. On the basis of eq 7, each intensity component decomposes into
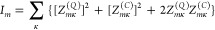
10such that σ_IR_ exhibits a molecular, σ_IR_^(*Q*)^, a cavity, σ_IR_^(*C*)^, and a mixed contribution, σ_IR_^(*X*)^. Finally, *Z*_*mκ*_ is determined by Cartesian components, *D*_00,κ_^(ec)^, of a generalized ground state dipole moment
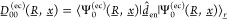
11which is evaluated with respect to the electron–photon ground state, |Ψ_0_^(ec)^⟩, where integration over electronic coordinates is indicated by .

We now introduce a systematic approximation to the CBO linear response approach^15^ based on cavity Born–Oppenheimer perturbation theory (CBO-PT).^19^ In CBO-PT, the light–matter interaction potential, , in eq 3 is treated as perturbation of the electronic subsystem^19^
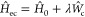
12with zeroth-order Hamiltonian  and formal perturbation parameter λ. In , the cavity potential, *V*_c_, is a constant with respect to the bare electronic problem, such that zeroth-order states are given by bare adiabatic electronic states, |Ψ_μ_^(e)^⟩. CBO-PT approximates nonresonant interactions between electrons and low-frequency cavity modes, i.e., electron–photon correlation, by perturbatively solving the electron–photon TISE (1).^19^ At the *n*th order of CBO-PT, i.e., CBO-PT(*n*), the approximate cPES, *E*_0_^(ec)^ ≈ ∑_*k*=0_^*n*^λ^*k*^*E*_0_^(*k*)^ (cf. section S1), provides access to the corresponding Hessian
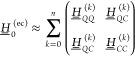
13with eigenvalues (Ω_*m*_^(*n*)^)^2^ and eigenvectors . In contrast to nonperturbative CBO linear response theory,^15^ we work directly with mass-weighted molecular normal modes with coordinates , which constitute the CBO-PT(0) reference. CBO-PT(*n*) linear vibro-polaritonic IR spectra are given by
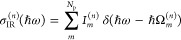
14with approximate vibro-polaritonic normal mode frequencies Ω_*m*_^(*n*)^ and IR intensities . Here, *Z*_*mκ*_^(*n*)^ = *Z*_*mκ*_^(*Q*,*n*)^ + *Z*_*mκ*_^(*C*,*n*)^ are approximate mode effective charges with (cf. section S1A)
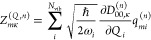
15
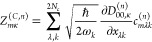
16which result from the linearized CBO-PT(*n*) ground-state dipole moment
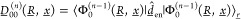
17where  is determined by |Φ_0_^(*n*–1)^⟩ = ∑_*k*=0_^*n*–1^λ^*k*^|Ψ_0_^(*k*)^⟩, which is the perturbatively corrected adiabatic ground state with |Ψ_0_^(*k*)^⟩ being defined in section S1. Note, in contrast to eq 7, both *Z*_*mκ*_^(*Q*,*n*)^ and *Z*_*mκ*_^(*C*,*n*)^ depend on prefactors with molecular normal mode, ω_*i*_, and cavity, ω_*k*_, frequencies that follow from the normal mode representation (cf. section S1A). Notably, CBO-PT relies exclusively on quantities that can be obtained from established *ab initio* quantum chemistry methods.

In the following, we provide explicit expressions up to perturbation order *n* ≤ 2, i.e., up to CBO-PT(2), which is sufficient to capture leading-order electron–photon correlation corrections.^19^ In CBO-PT(1), which does not account for electron–photon correlation, Hessian matrix elements read (cf. sections S1B and S1C)
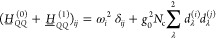
18
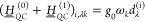
19
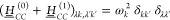
20The DSE term in eq 18 induces a normal mode frequency shift on the diagonal (*i* = *j*) and inter-normal mode (*i* ≠ *j*) couplings. The light–matter interaction matrix element in eq 19 contains a polarization-projected ground state dipole moment derivative, . The CBO-PT(1) IR spectrum, σ_IR_^(1)^, is fully determined by the molecular ground state dipole moment , leading to a first-order molecular IR intensity
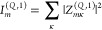
21
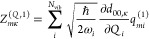
22since the cavity mode effective charge vanishes, *Z*_*mκ*_^(*C*,1)^ = 0, such that vibro-polaritonic contributions enter only via cavity-induced linear combinations of molecular normal modes. Thus, σ_IR_^(1)^, exclusively probes the effective matter response of the light–matter hybrid system under VSC.

Next, CBO-PT(2) Hessian matrix element corrections are given by (cf. sections S1D and S2)
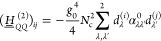
23
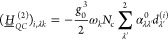
24
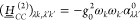
25which are all determined by polarization-projected static polarizability tensor elements . The leading-order correction (*g*_0_^2^) enters the cavity block in eq 25, which leads to a redshift of cavity mode frequencies and introduces a matter-mediated coupling of distinct cavity modes^19^ in agreement with nonperturbative results.^15^ The CBO-PT(2) IR spectrum, σ_IR_^(2)^(ℏω), is determined by second-order vibro-polaritonic frequencies, Ω_*m*_^(2)^, and intensities derived from second-order mode effective charges as
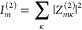
26
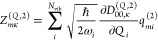
27
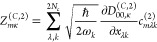
28which contains both molecular and cavity contributions with dipole derivatives explicitly given by (cf. section S3)
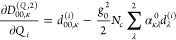
29
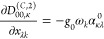
30

In both cases, we find static polarizability-dependent corrections with *D*_00,κ_^(*C*,2)^ being linear in *g*_0_ and *x*_*λk*_ (cf. section S3) in agreement with numerical results reported in the Supporting Information of ref (15). In contrast to CBO-PT(1), the nonvanishing CBO-PT(2) cavity intensity component allows us to address transmission spectra commonly measured in vibro-polaritonic chemistry,^4,7,8^ which exclusively probe the cavity response, σ_IR_^(*C*)^, of the light–matter hybrid system.

In the remainder of this work, we discuss electron–photon correlation effects in IR spectra of CO_2_ and Fe(CO)_5_ vibro-polaritonic models already studied in ref (15) as obtained from CBO-PT(2) linear response theory. We consider polarization-averaged IR spectra
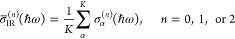
31to mimic the random molecular orientation, where the sum runs over all unique polarization components, α = *x*, *y*, or *z*, with *K* = 1, 2, or 3. Molecular structures, harmonic frequencies, dipole derivatives, and static polarizability tensor elements are obtained from density functional theory (DFT) using the TPSSh^25,26^ and B3LYP^27^ functionals and a Def2-TZVP basis set.^28,29^ Computational details are provided in section S4A. Spontaneous emission-induced Lorentzian peak broadening is assumed (we neglect other dissipative channels) with a full width at half-maximum κ = 41 cm^–1^ for a cavity quality factor  with experimentally motivated *Q* values of 50 for CO_2_ and 59 for Fe(CO)_5_^8,30,31^ (cf. section S4B).

We first discuss the antisymmetric stretching mode of CO_2_ under VSC with a single resonant cavity mode, *ℏω*_c_ = *ℏω*_as_ = 2400 cm^–1^ (TPSSh/Def2-TZVP), at coupling strength . Here, σ_IR_^(*n*)^(ℏω) = σ_*z*_^(*n*)^(ℏω) because only the dipole component along the molecular *z*-axis contributes. Figure 2a shows the lower and upper vibro-polaritonic transitions in CBO-PT(1) and CBO-PT(2) IR spectra with Rabi splittings, Ω_R_^(1)^ = 121 cm^–1^ and Ω_R_^(2)^ = 124 cm^–1^, which indicate a slightly stronger effective light–matter interaction in the presence of electron–photon correlation. In the CBO-PT(1) spectrum both vibro-polaritonic states exhibit nearly identical molecular and photonic contributions (cf. section S4B), in contrast to the CBO-PT(2) scenario, which is characterized by a dominantly photonic lower and dominantly molecular upper polariton state in agreement with nonperturbative CBO results.^15^ This asymmetry in state composition translates into a strongly asymmetric CBO-PT(2) spectrum in favor of the lower polariton transition, which is also found for larger values of the light–matter coupling parameter in ref (15), e.g., in Figure 2 there, equivalent to *g*_0_ employed here. (Note that a more quantitative comparison cannot be made because of differences in computational protocols and methods.) We will discuss peak asymmetry in more detail below. Turning to the details of σ_IR_^(2)^(ℏω) (Figure 2b), we compare molecular, σ_IR_^(*Q*,2)^, cavity, σ_IR_^(*C*,2)^, and mixed contributions, σ_IR_^(*X*,2)^ (cf. eq 10). Although σ_IR_^(*Q*,2)^ is dominant relative to σ_IR_^(*C*,2)^, σ_IR_^(*X*,2)^ determines the peak asymmetry of σ_IR_^(2)^ by “shifting” intensity from the upper to the lower vibro-polaritonic transition. This effect directly translates to the sign of the photonic contribution in linear combinations forming upper (+) and lower (−) polariton states (cf. section S4B). Mixed intensity contributions satisfy , because *Z*_∓κ_^(*C*,2)^ is negative due to the dipole derivative in eq 30. In addition, we recall that vibro-polaritonic IR transmission spectra probe the cavity response of the light–matter hybrid system,^4,7,8^ which translates here into the CBO-PT(2) cavity component, σ_IR_^(*C*,2)^, not accounted for in uncorrelated CBO-PT(1) models. In Figure 2c, we compare σ_IR_^(2)^ and σ_IR_^(*C*,2)^ normalized to the lower polariton intensity and find different relative intensities for upper polariton peaks. This observation can be traced back to the mixed component σ_IR_^(*X*,2)^ in σ_IR_^(2)^, which reduces the intensity of the upper polariton transition. Note that a larger frequency range (cf. section S4B) reveals in addition a purely molecular contribution of the CO_2_ bending modes at low frequencies of σ_IR_^(2)^, which is absent in σ_IR_^(*C*,2)^ displaying only vibro-polaritonic spectral features.

**Figure 2:**
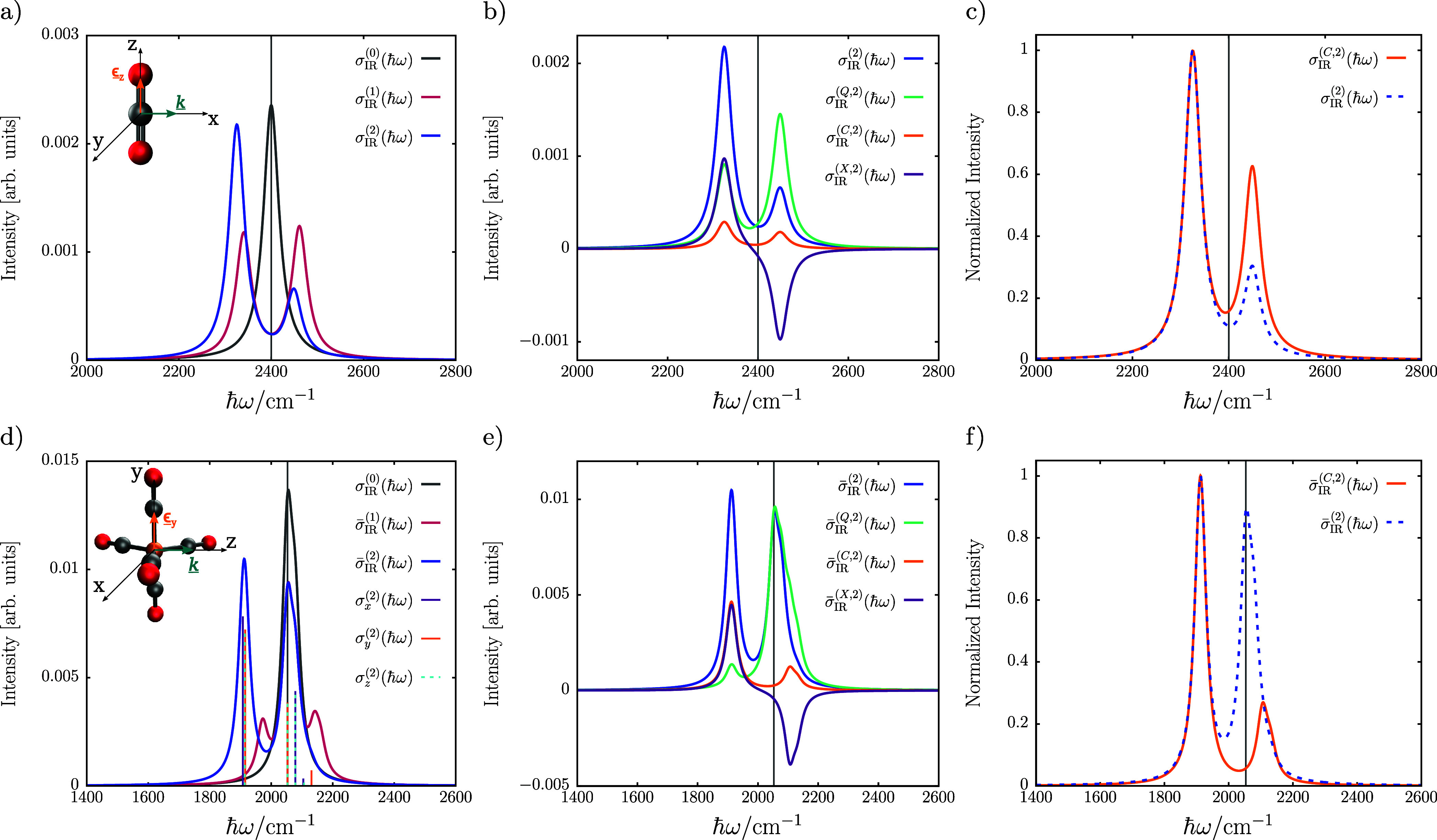
Linear vibro-polaritonic IR spectra for selected single-molecule models under VSC with a single cavity mode at coupling strength . The top row shows z-polarized IR spectra of the antisymmetric CO_2_ stretching mode under VSC with a single cavity mode, *ℏω*_c_ = *ℏω*_as_ = 2400 cm^–1^, with (a) CBO-PT(1) and CBO-PT(2) IR spectra, σ_IR_^(1)^(ℏω) and σ_IR_^(2)^(ℏω), in addition to the bare molecular spectrum, σ_IR_^(0)^(ℏω), (b) molecular, cavity, and mixed CBO-PT(2) contributions, σ_IR_^(*Q*,2)^(ℏω), σ_IR_^(*C*,2)^(ℏω), and σ_IR_^(*X*,2)^(ℏω), respectively, and (c) a comparison of normalized σ_IR_^(2)^(ℏω) and σ_IR_^(*C*,2)^(ℏω) (cf. the text for details). The bottom row shows polarization-averaged linear vibro-polaritonic IR spectra, , for the CO stretching band of a single Fe(CO)_5_ molecule under VSC with a single cavity mode, *ℏω*_c_ = *ℏω*_e′_ = 2052 cm^–1^, with panels d–f providing information analogous to that in panels a–c, respectively. Stick spectra, σ_α_^(2)^(ℏω), in panel d resemble individual polarization-dependent contributions to  in eq 31 with α = *x*, *y*, or *z* (cf. the text for details). The respective cavity mode frequency is indicated by a gray vertical line in all panels.

As a second example, we consider the CO stretching band of Fe(CO)_5_ under VSC, which is a both experimentally and theoretically relevant molecular multimode system.^8,15,33^ The CO stretching band contains a doubly degenerate equatorial normal mode (symmetry *e*′ in molecular point group *D*_3*h*_) and a single axial normal mode (*a*_2_^″^ in *D*_3*h*_) with harmonic frequencies *ℏω*_*e*′_ = 2052 cm^–1^ and  (TPSSh/Def2-TZVP, cf. section S4A)). We consider a single cavity mode tuned resonant to the equatorial *e*′ modes, *ℏω*_c_ = *ℏω*_*e*′_, at light–matter interaction . Here, *e*′ modes are (*x*, *z*)-polarized, while the *a*_2_^″^ mode is *y*-polarized (cf. Figures 1 and 2d). Thus, depending on the cavity mode polarization, either all molecular modes couple strongly to the cavity or some remain decoupled. In Figure 2d, we show polarization-averaged CBO-PT(1) and CBO-PT(2) IR spectra and the bare molecular CBO-PT(0) spectrum, where the average in eq 31 was performed with respect to polarization directions equivalent to the Cartesian components (α = *x*, *y*, or *z*). The latter are resolved by stick spectra, σ_α_^(2)^, for individual α values to highlight the polarization dependence of the different spectral contributions. Each σ_α_^(2)^ contains four peaks in the spectral region of interest due to the presence of *e*′ and *a*_2_^″^ modes in addition to the cavity mode. Note that the *a*_2_^″^ peak in σ_IR_^(0)^ is only visible as a blue-shifted shoulder relative to the dominant *e*′ transition for the herein chosen peak broadening. Under VSC, the effective *e*′ peak splits and we find in contrast to CO_2_ a pronounced molecular contribution related to uncoupled molecular modes (cf. σ_IR_^(0)^). The correlated CBO-PT(2) IR spectrum exhibits a strong redshift and significantly more intense vibro-polaritonic transitions relative to the uncorrelated CBO-PT(1) result, whereas both share a slight peak asymmetry in favor of the upper polariton peak. An analysis of  in terms of , and  reveals a trend similar to that of the CO_2_ example (cf. Figure 2e).  is the dominant contribution relative to , and the cross term again “shifts” the intensity from the upper to the lower vibro-polaritonic transition. Importantly, in contrast to the CO_2_ model,  contains here in addition to the matter response of the light–matter hybrid system bare molecular contributions, which relate to uncoupled CO stretching modes. A comparison of Rabi splittings, Ω_R_^(1)^ = 169 cm^–1^ and Ω_R_^(2)^ = 195 cm^–1^, where we extract the latter from  to avoid dominant molecular signatures, indicates also here a slightly enhanced effective light–matter interaction in the presence of electron–photon correlation. In Figure 2f, we compare  and  normalized relative to the lower polariton intensity. In contrast to the CO_2_ model, we find here a dominant  “upper” polariton contribution and a frequency mismatch of both peaks. Both observations relate to the fact that the “upper” polariton peak in  actually contains a dominant molecular contribution, which hides the vibro-polaritonic transition clearly visible in . Thus, correlation-corrected CBO-PT(2) IR spectra allow us to avoid artificial molecular intensity contributions in vibrational multimode systems under VSC, which are not probed in vibro-polaritonic transmission spectroscopy.^4,7,8^

The asymmetry in intensities and splittings can be understood from the CBO-PT(2) molecular normal and cavity mode frequencies in eqs 18 and 25. From a series expansion up to leading order in *g*_0_, one obtains dressed frequencies
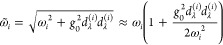
32
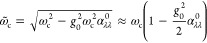
33where the dressed molecular frequency, , is slightly blue-shifted and the dressed cavity mode frequency, , is subject to a polarization-induced redshift. Thus, for the bare frequency resonance condition, ω_c_ = ω_*i*_, the CBO-PT(2) Hessian is in general not degenerate in the respective subspace, i.e., , which manifests in asymmetric intensities and/or splittings. A recent work^32^ proposed to replace ω_c_ = ω_*i*_ by a dressed resonance condition, , an idea already formulated in ref (15), which takes the matter feedback onto the vacuum cavity mode frequency, ω_c_, into account. Following this argument, we compare in Figure 3 CBO-PT IR spectra obtained from ω_c_ = ω_*i*_ with the cavity response, , calculated for a blue-detuned cavity frequency, *ℏω*_c_ + δ_c_ (dashed vertical line), such that  is satisfied. For a single CO_2_ molecule under VSC with *g*_0_ as before (Figure 3a), we observe a shift of δ_c_ = 30 cm^–1^ leading to nearly identical intensities for lower and upper polariton transitions in  in line with ref (32). The cavity response of Fe(CO)_5_ under VSC for the dressed resonance condition is obtained for , where we averaged  over different polarization-dependent δ_c_ values in analogy to IR spectra. For this multimode model, the peak asymmetry is also significantly reduced in , which in combination with the avoided molecular intensity contributions leads to a spectrum qualitatively closer to experimental results^8,33^ and highlights the potential relevance of feedback mechanisms between electrons and low-frequency cavity modes.

**Figure 3:**
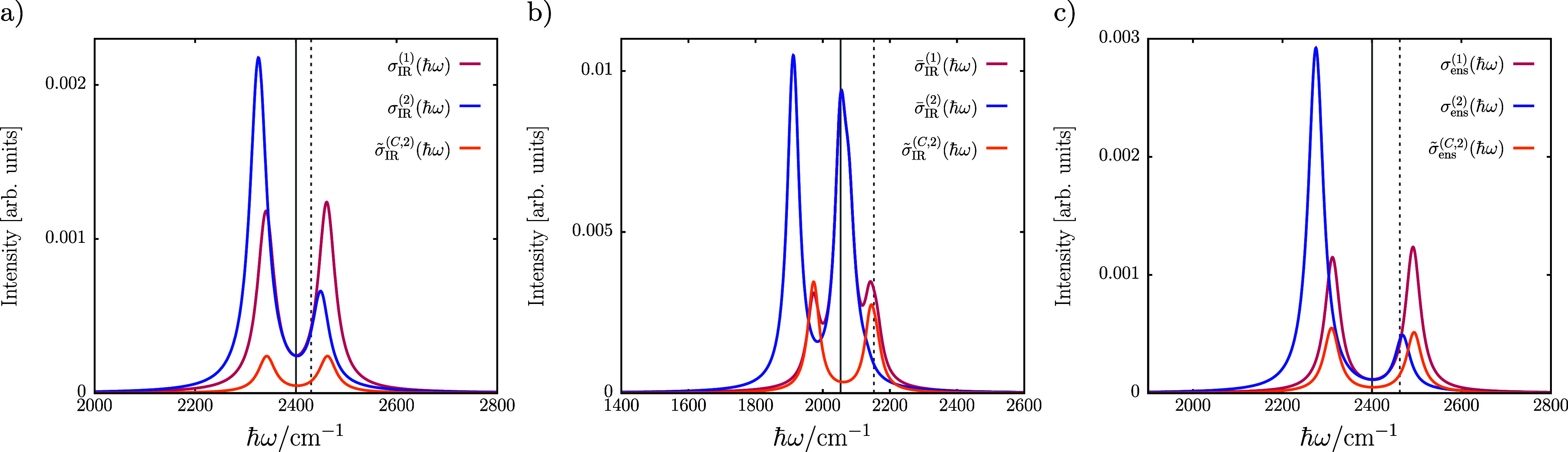
Effect of matter-induced cavity frequency renormalization on vibro-polaritonic transmission spectra, , with *ℏω*_c_ + δ_c_ (dashed gray vertical line in all panels) blue-detuned by δ_c_ from ω_*c*_ = ω_i_ (bold gray vertical line in all panels) such that . Shown is a comparison of  with σ_IR_^(1)^(ℏω) and σ_IR_^(2)^(ℏω) obtained from the bare resonance condition, ω_c_ = ω_*i*_, for (a) the single CO_2_ molecule under VSC with *ℏω*_c_ + δ_c_ = 2430 cm^–1^ and (b) the single Fe(CO)_5_ molecule under VSC with polarization-averaged  both at coupling strength  and (c) the explicit CO_2_ ensemble composed of *M* = 20 molecules with *ℏω*_c_ + δ_c_ = 2462 cm^–1^ at coupling strength .

We finally address collective strong coupling effects in two different molecular ensemble models of CO_2_ under VSC (cf. section S5): an explicit ensemble of *M* molecules in the dilute gas limit^34^ and an effective “ensemble” model, which contains a single CO_2_ molecule under VSC characterized by an effective enhanced interaction constant, .^15^ In Figure 4a, we compare ensemble and effective IR spectra, σ_ens_^(*n*)^ and σ_eff_^(*n*)^, respectively, for *M* = 20 parallel aligned CO_2_ molecules at  with the intensities being scaled by a factor of *M*^–1^ for reasons of comparison. In both cases, we find excellent agreement for σ_IR_^(1)^ and σ_IR_^(2)^. In addition, for the ensemble model, we observe qualitatively identical results for the different spectral contributions to σ_ens_^(2)^ in Figure 4b as in the single-molecule model in Figure 2. In addition, a comparison of normalized σ_ens_^(2)^ and σ_ens_^(*C*,2)^ for the explicit ensemble in Figure 4c reveals a slightly stronger mismatch of intensity ratios that can be observed from the upper polariton peak relative to Figure 2. Eventually, for the dressed resonance condition, we find here a required detuning of δ_c_ = 62 cm^–1^ for *M* = 20 molecules (cf. section S5), which leads to nearly symmetric intensities of upper and lower vibro-polaritonic transitions (cf. Figure 3c). Thus, approximate transmission spectra of molecular ensembles under VSC obtained from CBO-PT linear response theory can be calculated via effectively scaled single-molecule approaches subject to an effective light–matter interaction constant, , when intermolecular interactions are neglected in agreement with the nonperturbative CBO approach.^15^

**Figure 4:**
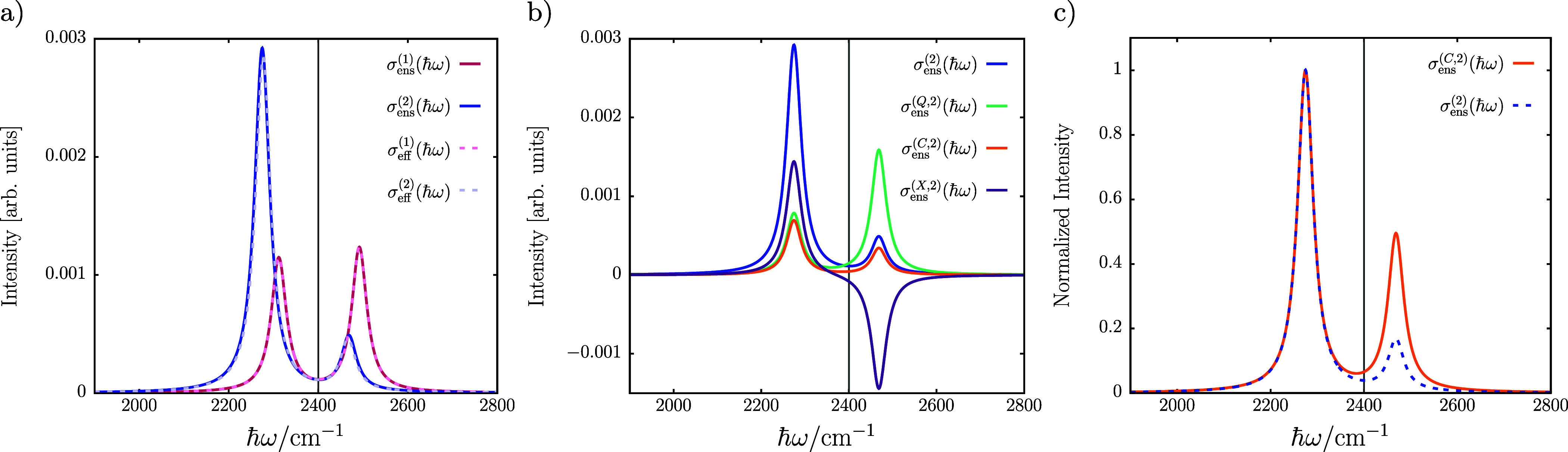
Linear vibro-polaritonic IR spectra for CO_2_ ensemble models under VSC with a single cavity mode, *ℏω*_c_ = *ℏω*_as_ = 2400 cm^–1^. (a) Ensemble and effective CBO-PT(*n*) IR spectra, σ_ens_^(*n*)^(ℏω) and σ_eff_^(*n*)^(ℏω) for *n* = 0 ,1, or 2. (b) Molecular, cavity, and mixed CBO-PT(2) contributions, σ_ens_^(*Q*,2)^(ℏω), σ_ens_^(*C*,2)^(ℏω), and σ_ens_^(*X*,2)^(ℏω), respectively, for the explicit ensemble. (c) Comparison of normalized σ_ens_^(2)^(ℏω) and σ_ens_^(*C*,2)^(ℏω) for the explicit ensemble. The respective cavity mode frequency is indicated by a gray vertical line.

In summary, we introduced a cavity Born–Oppenheimer perturbation theory (CBO-PT) linear response approach for calculating electron–photon correlation-corrected vibro-polaritonic IR spectra of polyatomic molecules under VSC. This approach approximates nonperturbative CBO linear response theory^15^ by perturbatively accounting for nonresonant interactions between electrons and low-frequency cavity modes, while fully relying on molecular *ab initio* quantum chemistry methods. Relative to the bare molecular zeroth-order IR spectrum, electron–photon correlation is accounted for at the second order of CBO-PT linear response theory. It manifests as a static polarizability-dependent cavity intensity component and related corrections of Hessian matrix elements, most notably a matter-induced cavity frequency shift and inter-cavity mode coupling. Those corrections capture the main characteristics of nonperturbative CBO linear response theory^15^ and provide additional physical insight due to their explicit relation to molecular and cavity mode properties. A comparison of uncorrelated CBO-PT(1) and correlation-corrected CBO-PT(2) IR spectra for CO_2_ and Fe(CO)_5_ vibro-polaritonic models reveals the impact of electron–photon correlation on vibro-polaritonic intensity ratios and Rabi splittings. In addition, CBO-PT(2) linear response theory allows to address the cavity response of the light–matter hybrid system related to experimentally relevant transmission spectroscopy. In both model scenarios, CBO-PT(2) linear response theory exhibits significant qualitative agreement with the nonperturbative CBO linear response results of ref (15). The origin of asymmetric intensities and splittings in CBO-PT(2) spectra was analyzed in context of a recently discussed dressed resonance condition,^32^ which takes into account the matter feedback on the bare cavity mode frequency and significantly reduces peak asymmetries also for molecular multimode and ensemble models. Finally, the CBO-PT linear response approach constitutes a promising since computationally easily accessible path to vibro-polaritonic IR spectra of polyatomic molecules under VSC, which accounts for nontrivial electron–photon correlation effects.

## Data Availability

The data that support the findings of this study are available from the corresponding author upon reasonable request.

## Supporting Information Available

Derivation of CBO-PT Hessian matrix elements, mode effective charges, and dipole moments and details of the computational methods for molecular properties and CBO-PT extension to molecular ensemble models in the dilute gas limit (PDF)

## References

[ref1] Ebbesen T. W. Hybrid light-matter states in a molecular and material science perspective. Acc. Chem. Res. 2016, 49, 2403 10.1021/acs.accounts.6b00295. 27779846

[ref2] Hirai K.; Hutchison J. A.; Uji-i H. Recent progress in vibropolaritonic chemistry. ChemPlusChem. 2020, 85, 1981 10.1002/cplu.202000411. 32869494

[ref3] Nagarajan K.; Thomas A.; Ebbesen T. W. Chemistry under vibrational strong coupling. J. Am. Chem. Soc. 2021, 143, 16877 10.1021/jacs.1c07420. 34609858

[ref4] Shalabney A.; George J.; Hutchison J. A.; Pupillo G.; Genet C.; Ebbesen T. W. Coherent coupling of molecular resonators with a microcavity mode. Nat. Commun. 2015, 6, 5981 10.1038/ncomms6981. 25583259 PMC4308833

[ref5] Shalabney A.; George J.; Hiura H.; Hutchison J. A.; Genet C.; Hellwig P.; Ebbesen T. W. Enhanced Raman Scattering from Vibro-Polariton Hybrid States. Angew. Chem., Int. Ed. 2015, 54, 7971–7975. 10.1002/anie.201502979. PMC4515085 26037542

[ref6] George J.; Shalabney A.; Hutchison J. A.; Genet C.; Ebbesen T. W. Liquid-phase vibrational strong coupling. J. Phys. Chem. Lett. 2015, 6, 1027 10.1021/acs.jpclett.5b00204. 26262864

[ref7] Long J. P.; Simpkins B. S. Coherent coupling between a molecular vibration and Fabry-Perot optical cavity to give hybridized states in the strong coupling limit. ACS Photonics 2015, 2, 130 10.1021/ph5003347.

[ref8] George J.; Chervy T.; Shalabney A.; Devaux E.; Hiura H.; Genet C.; Ebbesen T. W. Multiple Rabi Splittings under Ultrastrong Vibrational Coupling. Phys. Rev. Lett. 2016, 117, 153601 10.1103/PhysRevLett.117.153601. 27768350

[ref9] Chervy T.; Thomas A.; Akiki E.; Vergauwe R. M. A.; Shalabney A.; George J.; Devaux E.; Hutchison J. A.; Genet C.; Ebbesen T. W. Vibro-polaritonic IR emission in the strong coupling regime. ACS Photonics 2018, 5, 217 10.1021/acsphotonics.7b00677.

[ref10] Xiang B.; Ribeiro R. F.; Dunkelberger A. D.; Wang J.; Li Y.; Simpkins B. S.; Owrutsky J. C.; Yuen-Zhou J.; Xiong W. Two-dimensional infrared spectroscopy of vibrational polaritons. Proc. Natl. Acad. Sci. U. S. A. 2018, 115, 4845 10.1073/pnas.1722063115. 29674448 PMC5948987

[ref11] Li T. E.; Subotnik J. E.; Nitzan A. Cavity molecular dynamics simulations of liquid water under vibrational ultrastrong coupling. Proc. Natl. Acad. Sci. U. S. A. 2020, 117, 18324 10.1073/pnas.2009272117. 32680967 PMC7414078

[ref12] Fischer E. W.; Saalfrank P. Ground state properties and infrared spectra of anharmonic vibrational polaritons of small molecules in cavities. J. Chem. Phys. 2021, 154, 104311 10.1063/5.0040853. 33722029

[ref13] Lieberherr A. Z.; Furniss S. T. E.; Lawrence J. E.; Manolopoulos D. E. Vibrational strong coupling in liquid water from cavity molecular dynamics. J. Chem. Phys. 2023, 158, 234106 10.1063/5.0156808. 37326163

[ref14] Gómez J. A.; Vendrell O. Vibrational Energy Redistribution and Polaritonic Fermi Resonances in the Strong Coupling Regime. J. Phys. Chem. A 2023, 127, 1598 10.1021/acs.jpca.2c08608. 36758162

[ref15] Bonini J.; Flick J. Ab initio linear-response approach to vibro-polaritons in the cavity Born-Oppenheimer approximation. J. Chem. Theory Comput. 2022, 18, 2764 10.1021/acs.jctc.1c01035. 35404591 PMC9097282

[ref16] Schnappinger T.; Kowalewski M. Ab-Initio Vibro-Polaritonic Spectra in Strongly Coupled Cavity-Molecule Systems. J. Chem. Theory Comput. 2023, 19, 9278 10.1021/acs.jctc.3c01135. 38084914 PMC10753771

[ref17] Flick J.; Ruggenthaler M.; Appel H.; Rubio A. Atoms and molecules in cavities, from weak to strong coupling in quantum-electrodynamics (QED) chemistry. Proc. Natl. Acad. Sci. U.S.A. 2017, 114, 3026 10.1073/pnas.1615509114. 28275094 PMC5373338

[ref18] Flick J.; Appel H.; Ruggenthaler M.; Rubio A. Cavity Born-Oppenheimer approximation for correlated electron-nuclear-photon systems. J. Chem. Theory Comput. 2017, 13, 1616 10.1021/acs.jctc.6b01126. 28277664 PMC5390309

[ref19] Fischer E. W.; Saalfrank P. Beyond Cavity Born-Oppenheimer: On Nonadiabatic Coupling and Effective Ground State Hamiltonians in Vibro-Polaritonic Chemistry. J. Chem. Theory Comput. 2023, 19, 7215–7229. 10.1021/acs.jctc.3c00708. 37793029

[ref20] Galego J.; Climent C.; Garcia-Vidal F. J.; Feist J. Cavity Casimir-Polder forces and their effects in ground-state chemical reactivity. Phys. Rev. X 2019, 9, 021057 10.1103/PhysRevX.9.021057.

[ref21] Szidarovszky T. An efficient and flexible approach for computing rovibrational polaritons from first principles. J. Chem. Phys. 2023, 159, 014112 10.1063/5.0153293. 37409770

[ref22] Haugland T. S.; Philbin J. P.; Ghosh T. K.; Chen M.; Koch H.; Narang P. Understanding the polaritonic ground state in cavity quantum electrodynamics. arXiv 2023, 10.48550/arXiv.2307.14822. 40377188

[ref23] Schäfer C.; Fojt J.; Lindgren E.; Erhart P. Machine Learning for Polaritonic Chemistry: Accessing chemical kinetics. J. Am. Chem. Soc. 2024, 10.1021/jacs.3c12829. PMC10910569 38354223

[ref24] Interaction parameter *g*_0_ is equivalent to λ_c_ employed by Bonini and Flick.

[ref25] Tao J. M.; Perdew J. P.; Staroverov V. N.; Scuseria G. E. Climbing the density functional ladder: Nonempirical meta-generalized gradient approximation designed for molecules and solids. Phys. Rev. Lett. 2003, 91, 146401 10.1103/PhysRevLett.91.146401. 14611541

[ref26] Staroverov V. N.; Scuseria G. E.; Tao J.; Perdew J. P. Comparative assessment of a new nonempirical density functional: Molecules and hydrogen-bonded complexes. J. Chem. Phys. 2003, 119, 12129 10.1063/1.1626543. 28010100

[ref27] Becke A. D. Density-functional thermochemistry. III. The role of exact exchange. J. Chem. Phys. 1993, 98, 5648 10.1063/1.464913.

[ref28] Weigend F.; Ahlrichs R. Balanced basis sets of split valence, triple zeta valence and quadruple zeta valence quality for H to Rn: Design and assessment of accuracy. Phys. Chem. Chem. Phys. 2005, 7, 3297 10.1039/b508541a. 16240044

[ref29] Weigend F. Accurate Coulomb-fitting basis sets for H to Rn. Phys. Chem. Chem. Phys. 2006, 8, 1057 10.1039/b515623h. 16633586

[ref30] Ulusoy I. S.; Vendrell O. Dynamics and spectroscopy of molecular ensembles in a lossy microcavity. J. Chem. Phys. 2020, 153, 044108 10.1063/5.0011556. 32752693

[ref31] Fischer E. W.; Saalfrank P. Cavity-induced non-adiabatic dynamics and spectroscopy of molecular rovibrational polaritons studied by multi-mode quantum models. J. Chem. Phys. 2022, 157, 034305 10.1063/5.0098006. 35868933

[ref32] Fiechter M. R.; Richardson J. O. Understanding the Cavity Born-Oppenheimer Approximation. arXiv 2024, 10.48550/arXiv.2401.03532. 38717280

[ref33] Chen T.-T.; Du M.; Yang Z.; Yuen-Zhou J.; Xiong W. Cavity-enabled enhancement of ultrafast intramolecular vibrational redistribution over pseudorotation. Science 2022, 378, 790 10.1126/science.add0276. 36395241

[ref34] Sidler D.; Schnappinger T.; Obzhirov A.; Ruggenthaler M.; Kowalewski M.; Rubio A. Unravelling a Cavity Induced Molecular Polarization Mechanism from Collective Vibrational Strong Coupling. arXiv 2023, 10.48550/arXiv.2306.06004. PMC11103705 38717382

