# Supplementary material for: A Quantum Chemistry Approach to Linear Vibro-Polaritonic Infrared Spectra with Perturbative Electron–Photon Correlation
Source: J Phys Chem Lett. 2024 Feb 21;15(8):2262–9. doi: 10.1021/acs.jpclett.4c00105 (PMC10910601; doi:10.1021/acs.jpclett.4c00105)
Supplement: Supplementary file 1 — jz4c00105_si_001.pdf [file jz4c00105_si_001.pdf]

Supporting Information:
A Quantum Chemistry Approach to Linear Vibro-Polaritonic Infrared Spectra with Perturbative Electron-Photon Correlation

Eric W. Fischer,[1,2,*] Jan A. Syska,[2] and Peter Saalfrank[2,3,†]

[1] *Institut für Chemie, Humboldt-Universität zu Berlin, Brook-Taylor-Straße 2, D-12489 Berlin, Germany*
[2] *Institut für Chemie, Universität Potsdam, Karl-Liebknecht-Straße 24-25, D-14476 Potsdam-Golm, Germany*
[3] *Institut für Physik und Astronomie, Universität Potsdam, Karl-Liebknecht-Straße 24-25, D-14476 Potsdam-Golm, Germany*

(Dated: February 12, 2024)

We provide additional details on (i) the explicit derivation of approximate mode effective charges and vibro-polaritonic Hessian matrix elements up to CBO-PT(2), (ii) the role of the Franck-Condon approximation for CBO-PT(2) Hessian matrix elements, (iii) the derivation of a generalized CBO-PT(2) dipole moment and its derivatives, (iv) details on computational methods and vibro-polaritonic states and (v) a generalization of CBO-PT Hessians to molecular ensemble models under VSC.

S1. DERIVATION OF VIBRO-POLARITONIC MODE EFFECTIVE CHARGES AND HESSIAN MATRIX ELEMENTS

We explicitly derive approximate mode effective charges and vibro-polaritonic Hessian matrix elements for the CBO-PT(1) and CBO-PT(2) linear response approach. In order to simplify notation, we define polarization-projected permanent dipole derivatives and vertical transition dipole moments as

$$d^{(i)}_{\lambda k} = \left(\underline{e}_{\lambda k} \cdot \underline{d}^{(i)}_{00}\right) \quad , \quad d^{\mu}_{\lambda k} = \left(\underline{e}_{\lambda k} \cdot \underline{d}_{0\mu}\right) \quad , \tag{S1.1}$$

with $\underline{d}^{(i)}_{00} = \partial_{Q_i} \underline{d}_{00}|_{\underline{Q}=0}$ and $\underline{d}_{0\mu} = \langle \Psi^{(e)}_0 | \hat{\underline{d}}_{en} | \Psi^{(e)}_\mu \rangle_{\underline{r}}$ (molecular dipole operator $\hat{\underline{d}}_{en}$), as well as excitation energies between adiabatic states $|\Psi^{(e)}_0\rangle$ and $|\Psi^{(e)}_\mu\rangle$ as

$$\Delta^{(e)}_{0\mu} = E^{(e)}_0 - E^{(e)}_\mu \quad . \tag{S1.2}$$

Further, we rewrite the light-matter coupling constant of the kth-cavity mode as

$$g_k = \sqrt{\frac{\hbar\omega_k}{2\epsilon_0 V_{\mathrm{cav}}}} = \sqrt{\frac{\hbar\omega_k}{2}}\, g_0 \quad , g_0 = \frac{1}{\sqrt{\epsilon_0 V_{\mathrm{cav}}}} \quad , \tag{S1.3}$$

which allows us to express all terms via the universal coupling constant, g_0, which simply depends on the electric permittivity, ϵ_0, and the cavity volume, V_{cav}. Further, g_0 has units of $\sqrt{E_h}/(ea_0)$, since $[g_k] = E_h/(ea_0)$ and $[\sqrt{\hbar\omega_k}] = \sqrt{E_h}$. Alternatively, one directly obtains

$$[g_0] = \left[\frac{1}{\sqrt{\epsilon_0 V_{\mathrm{cav}}}}\right] = \frac{1}{\sqrt{\dfrac{e^2}{a_0 E_h}\, a_0^3}} = \frac{\sqrt{E_h}}{ea_0} \quad . \tag{S1.4}$$

The second-order energy in Rayleigh-Schrödinger perturbation theory is given by

$$E^{(ec)}_0 \approx \sum_{k=0}^{2} \lambda^k E^{(k)}_0 \quad , \tag{S1.5}$$

with

$$E^{(0)}_0 = E^{(e)}_0 + V_c \quad , \tag{S1.6}$$

$$E^{(1)}_0 = \langle \Psi^{(e)}_0 | \hat{W}_c | \Psi^{(e)}_0 \rangle_{\underline{r}} \quad , \tag{S1.7}$$

$$E^{(2)}_0 = \sum_{\mu\neq 0} \frac{|\langle \Psi^{(e)}_0 | \hat{W}_c | \Psi^{(e)}_\mu \rangle_{\underline{r}}|^2}{\Delta^{(e)}_{0\mu}} \quad , \tag{S1.8}$$

Further details are given in Sec.S1B-D below. At second-order in energy, first-order corrected states become relevant, which read

$$|\Phi^{(1)}_0\rangle = \sum_{k=0}^{1} \lambda^k |\Psi^{(k)}_0\rangle \quad , \tag{S1.9}$$

$$|\Psi^{(0)}_0\rangle = |\Psi^{(e)}_0\rangle \quad , \tag{S1.10}$$

$$|\Psi^{(1)}_0\rangle = \sum_{\mu\neq 0} \frac{\langle \Psi^{(e)}_\mu | \hat{W}_c | \Psi^{(e)}_0 \rangle_{\underline{r}}}{\Delta^{(e)}_{0\mu}} |\Psi^{(e)}_\mu\rangle \quad , \tag{S1.11}$$

with adiabatic electronic ground, $|\Psi^{(e)}_0\rangle$, and excited states, $|\Psi^{(e)}_\mu\rangle$. Further details are given in Sec.S3 below.

A. Vibro-Polaritonic Mode Effective Charges in Normal Mode Representation

We derive approximate mode effective charges in the normal mode representation. First, we linearize CBO-

* ericwfischer.sci@posteo.de
† peter.saalfrank@uni-potsdam.de

PT(n) permanent dipole moment components as

$$D^{(n)}_{00,\kappa} \approx D^{(0,n)}_{00,\kappa} + \sum_i^{N_{\mathrm{vib}}} \frac{\partial D^{(n)}_{00,\kappa}}{\partial Q_i} Q_i + \sum_{\lambda,k}^{2N_c} \frac{\partial D^{(n)}_{00,\kappa}}{\partial x_{\lambda k}} x_{\lambda k} \quad , \tag{S1.12}$$

with a constant term, $D^{(0,n)}_{00,\kappa}$, which will not contribute to vibro-polaritonic IR intensities. The m*th*-vibro-polaritonic transition in CBO-PT(n) is characterized by an intensity

$$I^{(n)}_m = \sum_\kappa |\langle 0|D^{(n)}_{00,\kappa}|m^{(n)}\rangle|^2 \quad , \tag{S1.13}$$

corresponding to a transition between vibro-polaritonic states, $|0\rangle$ and $|m^{(n)}\rangle$, which read in harmonic approximation

$$|0\rangle = |\underline{0}^{(Q)}, \underline{0}^{(C)}\rangle \quad , \tag{S1.14}$$

$$|m^{(n)}\rangle = \sum_j^{N_{\mathrm{vib}}} q^{(n)}_{mj} |1^{(Q)}_j\rangle + \sum_{\lambda',k'}^{2N_c} c^{(n)}_{m\lambda'k'} |1^{(C)}_{\lambda'k'}\rangle \quad . \tag{S1.15}$$

where $|m^{(n)}\rangle$ explicitly depends on the order n of CBO-PT(n). Here, $|0\rangle$ is the global ground state of all molecular normal and cavity modes, which is assumed to be not influenced under VSC. Further, the vibrational polariton, $|m^{(n)}\rangle$, resembles a linear combination of singly excited normal mode, $|1^{(Q)}_j\rangle$, and cavity mode states, $|1^{(C)}_{\lambda'k'}\rangle$, with expansion coefficients, $q^{(n)}_{mj}$ and $c^{(n)}_{m\lambda'k'}$, where our notation implicitly assume all remaining modes to reside in their respective ground states.

We now introduce the approximate mode effective charge for the linearized permanent dipole moment

$$Z^{(n)}_{m\kappa} = \langle 0|D^{(n)}_{00,\kappa}|m^{(n)}\rangle \tag{S1.16}$$

$$= \sum_i^{N_{\mathrm{vib}}} \frac{\partial D^{(n)}_{00,\kappa}}{\partial Q_i} \langle 0|Q_i|m^{(n)}\rangle \tag{S1.17}$$

$$+ \sum_{\lambda,k}^{2N_c} \frac{\partial D^{(n)}_{00,\kappa}}{\partial x_{\lambda k}} \langle 0|x_{\lambda k}|m^{(n)}\rangle \quad ,$$

which turns with the expansion of $|m^{(n)}\rangle$ into

$$Z^{(n)}_{m\kappa} = \sum_{i,j}^{N_{\mathrm{vib}}} \frac{\partial D^{(n)}_{00,\kappa}}{\partial Q_i} q^{(n)}_{mj} \langle 0|Q_i|1^{(Q)}_j\rangle \tag{S1.18}$$

$$+ \sum_{\lambda,k}^{2N_c} \sum_{\lambda',k'}^{2N_c} \frac{\partial D^{(n)}_{00,\kappa}}{\partial x_{\lambda k}} c^{(n)}_{m\lambda'k'} \langle 0|x_{\lambda k}|1^{(C)}_{\lambda'k'}\rangle \; .$$

Further, matrix elements between harmonic oscillator states evaluate to

$$\langle 0|Q_i|1^{(Q)}_j\rangle = \sqrt{\frac{\hbar}{2\omega_i}}\, \delta_{ij} \quad , \tag{S1.19}$$

$$\langle 0|x_{\lambda k}|1^{(C)}_{\lambda'k'}\rangle = \sqrt{\frac{\hbar}{2\omega_k}}\, \delta_{\lambda\lambda'}\delta_{kk'} \quad , \tag{S1.20}$$

which eventually leads to the CBO-PT(n) mode effective charge in normal mode representation

$$Z^{(n)}_{m\kappa} = Z^{(Q,n)}_{m\kappa} + Z^{(C,n)}_{m\kappa} \quad , \tag{S1.21}$$

with

$$Z^{(Q,n)}_{m\kappa} = \sum_i^{N_{\mathrm{vib}}} \sqrt{\frac{\hbar}{2\omega_i}} \frac{\partial D^{(n)}_{00,\kappa}}{\partial Q_i} q^{(n)}_{mi} \quad , \tag{S1.22}$$

$$Z^{(C,n)}_{m\kappa} = \sum_{\lambda,k}^{2N_c} \sqrt{\frac{\hbar}{2\omega_k}} \frac{\partial D^{(n)}_{00,\kappa}}{\partial x_{\lambda k}} c^{(n)}_{m\lambda k} \quad . \tag{S1.23}$$

B. CBO-PT(0) Hessian Matrix Elements

In harmonic approximation, the CBO-PT(0) cPES is given by

$$V^{(0)}(\underline{R}, \underline{x}) \approx V^{(e)}_0(\underline{Q}) + V_c(\underline{x}) \quad , \tag{S1.24}$$

$$= \sum_{i=1}^{N_{\mathrm{vib}}} \frac{\omega_i^2}{2} Q_i^2 + \sum_{\lambda,k}^{2N_c} \frac{\omega_k^2}{2} x_{\lambda k}^2 \quad . \tag{S1.25}$$

From here, the zeroth-order molecular and cavity block contributions follow immediately as

$$\left(\underline{\underline{H}}^{(0)}_{QQ}\right)_{ij} = \frac{\partial^2}{\partial Q_i \partial Q_j} V^{(e)}_0(\underline{Q}) = \omega_i^2\, \delta_{ij} \quad , \tag{S1.26}$$

$$\left(\underline{\underline{H}}^{(0)}_{CC}\right)_{\lambda k, \lambda' k'} = \frac{\partial^2 V_c(\underline{x})}{\partial x_{\lambda k} \partial x_{\lambda' k'}} = \omega_k^2\, \delta_{kk'}\delta_{\lambda\lambda'} \quad , \tag{S1.27}$$

$$\left(\underline{\underline{H}}^{(0)}_{QC}\right)_{i,\lambda k} = 0 \quad , \tag{S1.28}$$

with molecular normal mode frequencies, ω_i, and cavity mode frequencies, ω_k.

C. CBO-PT(1) Hessian Matrix Elements

The first-order CBO-PT Hessian is determined by the first-order perturbative energy correction

$$E^{(1)}_0(\underline{R}, \underline{x}) = \langle \Psi^{(e)}_0 | \hat{W}_c | \Psi^{(e)}_0 \rangle_{\underline{r}} = W_{00}(\underline{R}, \underline{x}) \quad , \tag{S1.29}$$

which explicitly reads

$$W_{00}(\underline{R}, \underline{x}) = g_0 \sum_{\lambda,k}^{2N_c} \omega_k\, d^0_{\lambda k} x_{\lambda k} + \frac{g_0^2}{2} \sum_{\lambda,k}^{2N_c} \sum_\alpha d^\alpha_{\lambda k} d^\alpha_{\lambda k} \, . \tag{S1.30}$$

In the second term, we introduced the resolution-of-the-identity (RI), $\sum_\alpha |\Psi^{(e)}_\alpha\rangle \langle \Psi^{(e)}_\alpha| = \mathbf{1}^{(e)}$, in the adiabatic electronic subspace between projected dipole moments of the DSE term. We now apply the Franck-Condon (FC) approximation

$$d^\alpha_{\lambda k}(\underline{R}) \approx d^\alpha_{\lambda k}(\underline{R}_0) = const. \quad , \quad \forall \alpha \neq 0 \quad , \tag{S1.31}$$

i.e., only vertical transition dipole moments are considered at the molecular reference configuration, $\underline{R}_0$. Then, we have

$$W_{00}(\underline{R},\underline{x}) = g_0 \sum_{\lambda,k}^{2N_c} \omega_k \, d^0_{\lambda k} x_{\lambda k} + \frac{g_0^2}{2} \sum_{\lambda,k}^{2N_c} d^0_{\lambda k} d^0_{\lambda k} + \frac{g_0^2}{2} \sum_{\lambda,k}^{2N_c} \sum_{\alpha \neq 0} d^\alpha_{\lambda k} d^\alpha_{\lambda k} \quad , \tag{S1.32}$$

where the last term depends only on the nuclear reference configuration due to the FC approximation and will therefore not contribute to the Hessian. We linearize the polarization-projected permanent dipole moment around the reference configuration (double-harmonic (DH) approximation)

$$d^0_{\lambda k} \approx d^{(0)}_{\lambda k} + \sum_{i}^{N_{\mathrm{vib}}} d^{(i)}_{\lambda k} \, Q_i \quad , \tag{S1.33}$$

with mass-weighted normal mode coordinate, Q_i, and $d^{(0)}_{\lambda k} = \underline{e}_{\lambda k} \cdot \underline{d}^{(0)}_{00}(\underline{R}_0)$. In DH- and FC-approximations, Hessian-relevant contributions in W_{00} reduce to

$$W_{00}(\underline{Q},\underline{x}) \approx g_0 \sum_{\lambda,k}^{2N_c} \omega_k \, x_{\lambda k} \sum_{i}^{N_{\mathrm{vib}}} d^{(i)}_{\lambda k} \, Q_i + \frac{g_0^2}{2} \sum_{\lambda,k}^{2N_c} \sum_{i,j}^{N_{\mathrm{vib}}} d^{(i)}_{\lambda k} \, d^{(j)}_{\lambda k} \, Q_i \, Q_j \quad . \tag{S1.34}$$

The molecular normal mode block is only determined by the DSE term ($\propto g_0^2$) due to its quadratic dependence on normal mode coordinates, such that

$$\begin{aligned} \left(\underline{\underline{H}}^{(1)}_{QQ}\right)_{ij} &= \frac{\partial^2}{\partial Q_i \partial Q_j} W_{00}(\underline{Q},\underline{x}) \quad , \\ &= \frac{g_0^2}{2} \sum_{\lambda,k}^{2N_c} \sum_{i',j'}^{N_{\mathrm{vib}}} d^{(i')}_{\lambda k} \, d^{(j')}_{\lambda k} \frac{\partial^2}{\partial Q_i \partial Q_j} Q_{i'} \, Q_{j'} \quad , \\ &= g_0^2 \sum_{\lambda,k}^{2N_c} d^{(i)}_{\lambda k} \, d^{(j)}_{\lambda k} \quad , \\ &= g_0^2 N_c \sum_{\lambda}^{2} d^{(i)}_{\lambda} d^{(j)}_{\lambda} \quad , \end{aligned} \tag{S1.35}$$

where we obtained the third line via the identity

$$\begin{aligned} \frac{\partial^2}{\partial Q_i \partial Q_j} Q_{i'} Q_{j'} &= \frac{\partial Q_{i'}}{\partial Q_j} \frac{\partial Q_{j'}}{\partial Q_i} + \frac{\partial Q_{i'}}{\partial Q_i} \frac{\partial Q_{j'}}{\partial Q_j} \quad , &(S1.36) \\ &= \delta_{i'j} \delta_{j'i} + \delta_{i'i} \delta_{j'j} \quad , &(S1.37) \end{aligned}$$

where an additional factor two results from the fact, that both Kronecker delta products provide the same term. The forth line follows from the assumption of identical polarization vectors for all cavity modes

$$\sum_{\lambda,k}^{2N_c} d^{(i)}_{\lambda k} \, d^{(j)}_{\lambda k} = N_c \sum_{\lambda}^{2} d^{(i)}_{\lambda} d^{(j)}_{\lambda} \quad , \tag{S1.38}$$

which allows us to perform the sum over mode index k explicitly. Next, the interaction matrix elements follow as

$$\begin{aligned} \left(\underline{\underline{H}}^{(1)}_{QC}\right)_{i,\lambda k} &= \frac{\partial^2}{\partial Q_i \partial x_{\lambda k}} W_{00}(\underline{Q},\underline{x}) \quad , &(S1.39) \\ &= g_0 \, \omega_k \, d^{(i)}_{\lambda k} \quad , &(S1.40) \end{aligned}$$

where we treat, (λk), as a block-matrix index, such that every k specifies a corresponding 2×2-subblock and λ addresses related subblock elements. Finally, we have

$$\left(\underline{\underline{H}}^{(1)}_{CC}\right)_{\lambda k, \lambda' k'} = 0 \quad . \tag{S1.41}$$

D. CBO-PT(2) Hessian Matrix Elements

The CBO-PT(2) Hessian is determined by the corresponding energy correction

$$E^{(2)}_0(\underline{R},\underline{x}) = \sum_{\mu \neq 0} \frac{|W_{\mu 0}|^2}{\Delta^{(e)}_{0\mu}} \quad , \tag{S1.42}$$

where the matrix element, $W_{\mu 0}$, follows in DH-/FC-approximations as

$$W_{0\mu}(\underline{Q},\underline{x}) \approx g_0 \sum_{\lambda,k}^{2N_c} \omega_k \, d^\mu_{\lambda k} \, x_{\lambda k} + \frac{g_0^2}{2} \sum_{\lambda,k}^{2N_c} d^\mu_{\lambda k} \sum_{i}^{N_{\mathrm{vib}}} d^{(i)}_{\lambda k} \, Q_i \, . \tag{S1.43}$$

Here, all constant or linear terms, which do not contribute to the Hessian, were neglected. Further, we consider only vertical excitation energies in FC approximation

$$\Delta^{(e)}_{0\mu}(\underline{R}) \approx \Delta^{(e)}_{0\mu}(\underline{R}_0) = const. \quad . \tag{S1.44}$$

Starting with the molecular contribution as determined by the squared second term of Eq.(S1.43), we find

$$\begin{aligned} \left(\underline{\underline{H}}^{(2)}_{QQ}\right)_{ij} &= \sum_{\mu \neq 0} \frac{1}{\Delta^{(e)}_{0\mu}} \frac{\partial^2}{\partial Q_i \partial Q_j} W_{0\mu} W_{\mu 0} \quad , \\ &= \frac{g_0^4}{4} \sum_{\mu \neq 0} \frac{1}{\Delta^{(e)}_{0\mu}} \sum_{i',j'}^{N_{\mathrm{vib}}} \sum_{\lambda,k}^{2N_c} d^\mu_{\lambda k} d^{(i')}_{\lambda k} \\ &\quad \times \sum_{\lambda',k'}^{2N_c} d^\mu_{\lambda' k'} d^{(j')}_{\lambda' k'} \frac{\partial^2}{\partial Q_i \partial Q_j} Q_{i'} Q_{j'} \quad , \\ &= \frac{g_0^4}{2} \sum_{\lambda,k}^{2N_c} d^{(i)}_{\lambda k} \sum_{\lambda',k'}^{2N_c} d^{(j)}_{\lambda' k'} \sum_{\mu \neq 0} \frac{d^\mu_{\lambda k} \, d^\mu_{\lambda' k'}}{\Delta^{(e)}_{0\mu}} \quad , \end{aligned} \tag{S1.45}$$

where we took into account the FC approximation in the first line, *i.e.*, derivatives do not act on *vertical* excitation energies, $\Delta^{(e)}_{0\mu}$, and used identity Eq.(S1.37) in the third line.

Further, twice the cross term in Eq.(S1.43) provide corrections to the normal mode-cavity coupling block as

$$\begin{aligned}\left(\underline{\underline{H}}^{(2)}_{QC}\right)_{i,\lambda k} &= \sum_{\mu\neq 0}\frac{1}{\Delta^{(e)}_{0\mu}}\frac{\partial^2}{\partial Q_i \partial x_{\lambda k}} W_{0\mu}W_{\mu 0} \quad , \\ &= g_0^3 \sum_{\mu\neq 0}\frac{1}{\Delta^{(e)}_{0\mu}}\sum_{\lambda',k'}^{2N_c} d^{\mu}_{\lambda' k'}\sum_{i'}^{N_\mathrm{vib}} d^{(i')}_{\lambda' k'} \\ &\quad \times \sum_{\sigma,l}^{2N_c}\omega_l\, d^{\mu}_{\sigma l}\frac{\partial^2}{\partial Q_i \partial x_{\lambda k}} x_{\sigma l}\, Q_{i'} \quad , \\ &= g_0^3\,\omega_k \sum_{\lambda',k'}^{2N_c} d^{(i)}_{\lambda' k'}\sum_{\mu\neq 0}\frac{d^{\mu}_{\lambda k}\, d^{\mu}_{\lambda' k'}}{\Delta^{(e)}_{0\mu}} \quad , \end{aligned} \tag{S1.46}$$

and the cavity block finally results from the squared first term of Eq.(S1.43) as

$$\begin{aligned}\left(\underline{\underline{H}}^{(2)}_{CC}\right)_{\lambda k,\lambda' k'} &= \sum_{\mu\neq 0}\frac{1}{\Delta^{(e)}_{0\mu}}\frac{\partial^2}{\partial x_{\lambda k} \partial x_{\lambda' k'}} W_{0\mu}W_{\mu 0} \quad , \\ &= g_0^2 \sum_{\mu\neq 0}\frac{1}{\Delta^{(e)}_{0\mu}}\sum_{\sigma,l}^{2N_c}\omega_l\, d^{\mu}_{\sigma l} \\ &\quad \times \sum_{\sigma',l'}^{2N_c}\omega_{l'}\, d^{\mu}_{\sigma' l'}\frac{\partial^2}{\partial x_{\lambda k} \partial x_{\lambda' k'}} x_{\sigma l}\, x_{\sigma' l'} \quad , \\ &= 2 g_0^2\,\omega_k\,\omega_{k'} \sum_{\mu\neq 0}\frac{d^{\mu}_{\lambda k}\, d^{\mu}_{\lambda' k'}}{\Delta^{(e)}_{0\mu}} \quad , \end{aligned} \tag{S1.47}$$

where we used a slightly more general variant of Eq.(S1.37) in the third line

$$\begin{aligned}\frac{\partial^2}{\partial x_{\lambda k}\partial x_{\lambda' k'}} x_{\sigma l} x_{\sigma' l'} &= \frac{\partial x_{\sigma l}}{\partial x_{\lambda k}}\frac{\partial x_{\sigma' l'}}{\partial x_{\lambda' k'}} + \frac{\partial x_{\sigma' l'}}{\partial x_{\lambda k}}\frac{\partial x_{\sigma l}}{\partial x_{\lambda' k'}} \,, \\ &= \delta_{\sigma\lambda}\,\delta_{lk}\,\delta_{\sigma'\lambda'}\,\delta_{l'k'} + \delta_{\sigma'\lambda}\,\delta_{l'k}\,\delta_{\sigma\lambda'}\,\delta_{lk'} \,. \end{aligned} \tag{S1.48}$$

E. Static Polarizability Formulation of CBO-PT(2)

The static polarizability tensor, $\underline{\underline{\alpha}}_0$, is defined by elements[2]

$$\alpha^0_{ab} = -2\sum_{\mu\neq 0}\frac{d^{\mu}_a\, d^{\mu}_b}{\Delta^{(e)}_{0\mu}} \quad , \tag{S1.49}$$

which we can exploit to rewrite CBO-PT(2) Hessian matrix elements above. Since elements, α^0_{ab}, can be obtained from linear response theory, Eq.(S1.49) allows for circumventing the unfavourable convergence properties of the sum-over-states expression.[2] Further, since polarization-projected transition dipole moments in CBO-PT(2) Hessian matrix elements do not differ for different cavity mode indices, k, we can introduce polarization-projected static polarizability tensor elements as

$$\sum_{\mu\neq 0}\frac{d^{\mu}_{\lambda}\, d^{\mu}_{\lambda'}}{\Delta^{(e)}_{0\mu}} = -\frac{\alpha^0_{\lambda\lambda'}}{2} \quad , \tag{S1.50}$$

such that CBO-PT(2) Hessian matrix elements can be compactly expressed as

$$\left(\underline{\underline{H}}^{(2)}_{QQ}\right)_{ij} = -\frac{g_0^4}{4} N_c^2 \sum_{\lambda,\lambda'}^{2} d^{(i)}_{\lambda}\,\alpha^0_{\lambda\lambda'}\, d^{(j)}_{\lambda'} \quad , \tag{S1.51}$$

$$\left(\underline{\underline{H}}^{(2)}_{QC}\right)_{i,\lambda k} = -\frac{g_0^3}{2}\omega_k\, N_c \sum_{\lambda'}^{2} \alpha^0_{\lambda\lambda'}\, d^{(i)}_{\lambda'} \quad , \tag{S1.52}$$

$$\left(\underline{\underline{H}}^{(2)}_{CC}\right)_{\lambda k,\lambda' k'} = -g_0^2\,\omega_k\,\omega_{k'}\,\alpha^0_{\lambda\lambda'} \quad . \tag{S1.53}$$

S2. DETAILS ON FRANCK-CONDON APPROXIMATION

We demonstrate that the Franck-Condon approximation is equivalent to neglecting corrections of CBO-PT(2) Hessian molecular (QQ) and light-matter interaction (QC) blocks, which scale as $(\Delta^{(e)}_{0\mu})^{-2}$ and $(\Delta^{(e)}_{0\mu})^{-3}$, respectively. For now, we assume excitation energies, $\Delta^{(e)}_{0\mu}$, in the second-order correction, $E^{(2)}_0$, to depend on normal mode coordinates. The corresponding first derivative follows as

$$\frac{\partial}{\partial Q_i}\frac{W_{0\mu}}{\Delta^{(e)}_{0\mu}} = \frac{(\partial_{Q_i} W_{0\mu})}{\Delta^{(e)}_{0\mu}} - \frac{W_{0\mu}\left(\partial_{Q_i}\Delta^{(e)}_{0\mu}\right)}{(\Delta^{(e)}_{0\mu})^2} \quad , \tag{S2.1}$$

and the second derivative takes the form

$$\begin{aligned}\frac{\partial}{\partial Q_i \partial Q_j}\frac{W_{0\mu}}{\Delta^{(e)}_{0\mu}} &= \frac{(\partial_{Q_i}\partial_{Q_j} W_{0\mu})}{\Delta^{(e)}_{0\mu}} - \frac{(\partial_{Q_i} W_{0\mu})\left(\partial_{Q_j}\Delta^{(e)}_{0\mu}\right)}{(\Delta^{(e)}_{0\mu})^2} \\ &\quad - \frac{(\partial_{Q_j} W_{0\mu})\left(\partial_{Q_i}\Delta^{(e)}_{0\mu}\right)}{(\Delta^{(e)}_{0\mu})^2} - \frac{W_{0\mu}\left(\partial_{Q_i}\partial_{Q_j}\Delta^{(e)}_{0\mu}\right)}{(\Delta^{(e)}_{0\mu})^2} \\ &\quad + \frac{2\,W_{0\mu}\left(\partial_{Q_i}\Delta^{(e)}_{0\mu}\right)\left(\partial_{Q_j}\Delta^{(e)}_{0\mu}\right)}{(\Delta^{(e)}_{0\mu})^3} \quad , \end{aligned} \tag{S2.2}$$

with $\frac{\partial}{\partial Q_i} = \partial_{Q_i}$. In the FC approximation, we approximate excitation energies to be independent of normal mode coordinates, *i.e.*, being only *vertical*, with

$$\partial_{Q_i}\Delta^{(e)}_{0\mu} \approx 0 \quad , \tag{S2.3}$$

such that all terms in Eq.(S2.2) besides the first one vanish identically. Thus, contributions proportional to $(\Delta^{(e)}_{0\mu})^{-2}$ and $(\Delta^{(e)}_{0\mu})^{-3}$ are neglected, such that the CBO-PT(2) Hessian is accurate up to $(\Delta^{(e)}_{0\mu})^{-1}$ in line with the CBO-PT(2) cPES correction.

S3. DERIVATION OF CBO-PT(2) PERMANENT DIPOLE DERIVATIVES

We derive explicit expressions for CBO-PT(2) permanent dipole derivatives. Starting with the CBO-PT(2) generalized permanent dipole moment

$$\underline{D}^{(2)}_{00}(\underline{R},\underline{x}) = \langle \Phi^{(1)}_0(\underline{R},\underline{x}) | \hat{\underline{d}}_{en} | \Phi^{(1)}_0(\underline{R},\underline{x}) \rangle \quad , \tag{S3.1}$$

which is determined by the first-order corrected adiabatic state

$$|\Phi^{(1)}_0\rangle = |\Psi^{(e)}_0\rangle + |\Psi^{(1)}_0\rangle \quad , \tag{S3.2}$$

$$= |\Psi^{(e)}_0\rangle + \sum_{\mu\neq 0} \frac{W_{0\mu}}{\Delta^{(e)}_{0\mu}} |\Psi^{(e)}_\mu\rangle \quad , \tag{S3.3}$$

we find

$$\underline{D}^{(2)}_{00}(\underline{R},\underline{x}) = \langle \Psi^{(e)}_0 | \hat{\underline{d}}_{en} | \Psi^{(e)}_0 \rangle_{\underline{r}} + 2\,\langle \Psi^{(e)}_0 | \hat{\underline{d}}_{en} | \Psi^{(1)}_0 \rangle_{\underline{r}} + \langle \Psi^{(1)}_0 | \hat{\underline{d}}_{en} | \Psi^{(1)}_0 \rangle_{\underline{r}} \quad . \tag{S3.4}$$

In DH- and FC-approximations, the given matrix elements read

$$\langle \Psi^{(e)}_0 | \hat{\underline{d}}_{en} | \Psi^{(e)}_0 \rangle_{\underline{r}} = \underline{d}^{(0)}_{00} + \sum_i^{N_\mathrm{vib}} \underline{d}^{(i)}_{00}\, Q_i \quad , \tag{S3.5}$$

$$2\,\langle \Psi^{(e)}_0 | \hat{\underline{d}}_{en} | \Psi^{(1)}_0 \rangle_{\underline{r}} = 2 \sum_{\mu\neq 0} \frac{\underline{d}_{0\mu}}{\Delta^{(e)}_{0\mu}} W_{0\mu} \quad , \tag{S3.6}$$

$$\langle \Psi^{(1)}_0 | \hat{\underline{d}}_{en} | \Psi^{(1)}_0 \rangle_{\underline{r}} = \sum_{\mu,\mu'\neq 0} \frac{W_{0\mu}\, \underline{d}_{\mu\mu'}\, W_{\mu' 0}}{\Delta^{(e)}_{0\mu}\, \Delta^{(e)}_{0\mu'}} \quad . \tag{S3.7}$$

In the following, we consider only leading order corrected IR intensities obtain from Eqs.(S3.5) and (S3.6) and neglect higher-order contributions of Eq.(S3.7), such that

$$\underline{D}^{(2)}_{00} \approx \langle \Psi^{(e)}_0 | \hat{\underline{d}}_{en} | \Psi^{(e)}_0 \rangle_{\underline{r}} + 2\,\langle \Psi^{(e)}_0 | \hat{\underline{d}}_{en} | \Psi^{(1)}_0 \rangle_{\underline{r}} \quad . \tag{S3.8}$$

The cross term in Eq.(S3.6) is explicitly given by

$$2\,\langle \Psi^{(e)}_0 | \hat{\underline{d}}_{en} | \Psi^{(1)}_0 \rangle_{\underline{r}} = 2 g_0 \sum_{\lambda,k}^{2N_c} \omega_k\, x_{\lambda k} \sum_{\mu\neq 0} \frac{d^{\mu}_{\lambda k}\, \underline{d}_{0\mu}}{\Delta^{(e)}_{0\mu}} + g_0^2 \sum_{\lambda,k}^{2N_c} \sum_i^{N_\mathrm{vib}} d^{(i)}_{\lambda k}\, Q_i \sum_{\mu\neq 0} \frac{d^{\mu}_{\lambda k}\, \underline{d}_{0\mu}}{\Delta^{(e)}_{0\mu}} \quad , \tag{S3.9}$$

$$= -g_0 \sum_{\lambda,k}^{2N_c} \omega_k\, \underline{\alpha}^0_\lambda x_{\lambda k} - \frac{g_0^2}{2} \sum_{\lambda,k}^{2N_c} \underline{\alpha}^0_\lambda \sum_i^{N_\mathrm{vib}} d^{(i)}_{\lambda k}\, Q_i \quad , \tag{S3.10}$$

where we used the identity

$$\sum_{\mu\neq 0} \frac{d^{\mu}_{\lambda k}\, \underline{d}_{0\mu}}{\Delta^{(e)}_{0\mu}} = -\frac{\underline{\alpha}^0_\lambda}{2} \quad . \tag{S3.11}$$

Thus, the linearized CBO-PT(2) dipole moment can be written as

$$\underline{D}^{(2)}_{00} = \underline{D}^{(Q,2)}_{00} + \underline{D}^{(C,2)}_{00} \quad , \tag{S3.12}$$

and contains a molecular term with cartesian components ($\kappa = x, y, z$)

$$D^{(Q,2)}_{00,\kappa}(\underline{Q}) = d^{(0)}_{00,\kappa} + \sum_i^{N_\mathrm{vib}} d^{(i)}_{00,\kappa}\, Q_i - \frac{g_0^2}{2} N_c \sum_\lambda^{2} \alpha^0_{\kappa\lambda} \sum_i^{N_\mathrm{vib}} d^{(i)}_\lambda Q_i \quad , \tag{S3.13}$$

as well as a cavity component

$$D^{(C,2)}_{00,\kappa}(\underline{x}) = -g_0 \sum_{\lambda,k}^{2N_c} \omega_k\, \alpha^0_{\kappa\lambda}\, x_{\lambda k} \quad . \tag{S3.14}$$

The normal-mode derivative of the molecular contribution follows as

$$\frac{\partial D^{(Q,2)}_{00,\kappa}}{\partial Q_i} = d^{(i)}_{00,\kappa} - \frac{g_0^2}{2} N_c \sum_\lambda^{2} \alpha^0_{\kappa\lambda}\, d^{(i)}_\lambda \quad , \tag{S3.15}$$

and the cavity coordinate derivative is given by

$$\frac{\partial D^{(C,2)}_{00,\kappa}}{\partial x_{\lambda k}} = -g_0\, \omega_k\, \alpha^0_{\kappa\lambda} \quad . \tag{S3.16}$$

S4. COMPUTATIONAL AND VIBRO-POLARITONIC DETAILS

A. Computational Details

Molecular equilibrium structures of CO_2 and $Fe(CO)_5$ have been optimized via TPSSh/Def2TZVP with a subsequent normal mode analysis providing normal mode frequencies and permanent dipole derivatives. Static polarizability tensor elements were obtained via CAM-B3LYP/Def2TZVP for both molecules. All calculations were performed with Gaussian16.[4] Optimized equilibrium structures are provided in Tab.I. Dipole derivatives for the asymmetric CO_2 stretching mode (TPSSh/Def2TZVP) and non-vanishing static-polarizability tensor elements for CO_2 (CAM-B3LYP/Def2TZVP) are given in Tab.II. Axis are given with respect to optimized equilibrium structures. Same properties for equatorial e'- and axial a''_2-modes of $Fe(CO)_5$ are given in Tab.III.

Vibro-polaritonic IR spectra with Lorentzian peak broadening are evaluated as

$$\sigma^{(n)}_\mathrm{IR}(\hbar\omega) = \sum_m^{N_p} I^{(n)}_m \frac{\kappa}{2\pi} \frac{1}{(\hbar\omega - \hbar\Omega^{(n)}_m)^2 + \frac{\kappa^2}{4}} \quad , \tag{S4.1}$$

for a frequency interval, $\hbar\omega \in [0, 3600]\mathrm{cm}^{-1}$, discretized by $N_\omega = 4000$ equidistant grid points and κ given in the main text.

TABLE I. Cartesian coordinates for CO_2 and $Fe(CO)_5$ equilibrium structures optimized on TPSSh/Def2TZVP level of theory.

Atom	X	Y	Z
O	0.000000	-0.000000	0.386715
C	0.000000	-0.000000	1.550000
O	0.000000	-0.000000	2.713285
Fe	0.000000	0.000000	0.002909
C	-0.000000	0.000000	1.809744
C	1.811959	-0.000000	0.001959
C	0.000000	1.565109	-0.899762
C	-0.000000	-1.565109	-0.899762
C	-1.811959	0.000000	0.001959
O	-0.000000	0.000000	2.955158
O	0.000000	2.557516	-1.471811
O	-0.000000	-2.557516	-1.471811
O	2.953719	-0.000000	0.000929
O	-2.953719	0.000000	0.000929

TABLE II. Dipole derivatives for the asymmetric CO_2 stretching mode (TPSSh/Def2TZVP) and non-vanishing static-polarizability tensor elements for CO_2 (CAM-B3LYP/Def2TZVP). All values in atomic units.

$\partial d_{00,x}/\partial Q_{\mathrm{as}}$	$\partial d_{00,y}/\partial Q_{\mathrm{as}}$	$\partial d_{00,z}/\partial Q_{\mathrm{as}}$
9.9e-16	-5.6e-17	1.8e-02
α^0_{xx}	α^0_{yy}	α^0_{zz}
10.2319	10.2319	24.75

TABLE III. Dipole derivatives for equatorial e'- and axial a_2''-modes of $Fe(CO)_5$ (TPSSh/Def2TZVP) and non-vanishing static-polarizability tensor elements for $Fe(CO)_5$ (CAM-B3LYP/Def2TZVP) (Gaussian16). All values in atomic units.

$\partial d_{00,x}/\partial Q_{\mathrm{e}'}$	$\partial d_{00,y}/\partial Q_{\mathrm{e}'}$	$\partial d_{00,z}/\partial Q_{\mathrm{e}'}$
2.64e-02	-6.7e-10	-5.1e-09
-3.8e-11	-4.5e-09	-1.4e-04
$\partial d_{00,x}/\partial Q_{\mathrm{a}_2''}$	$\partial d_{00,y}/\partial Q_{\mathrm{a}_2''}$	$\partial d_{00,z}/\partial Q_{\mathrm{a}_2''}$
9.9e-16	-5.6e-17	1.8e-02
α^0_{xx}	α^0_{yy}	α^0_{zz}
103.729	95.1879	95.1267

B. Details of Vibro-Polaritonic States

In harmonic oscillator systems, upper (+) and lower (-) vibro-polaritonic states resemble symmetric and antisymmetric linear combinations of singly excited molecular vibrational or cavity mode states as given by

$$|\pm^{(n)}\rangle = a_{\pm}^{(n)}\,|1_{\mathrm{as}}, 0_c\rangle \pm b_{\pm}^{(n)}\,|0_{\mathrm{as}}, 1_c\rangle \quad , \qquad \text{(S4.2)}$$

with single excited normal and cavity mode states, $|1_{\mathrm{as}}, 0_c\rangle$ and $|0_{\mathrm{as}}, 1_c\rangle$, here illustratively for the CO_2 model.

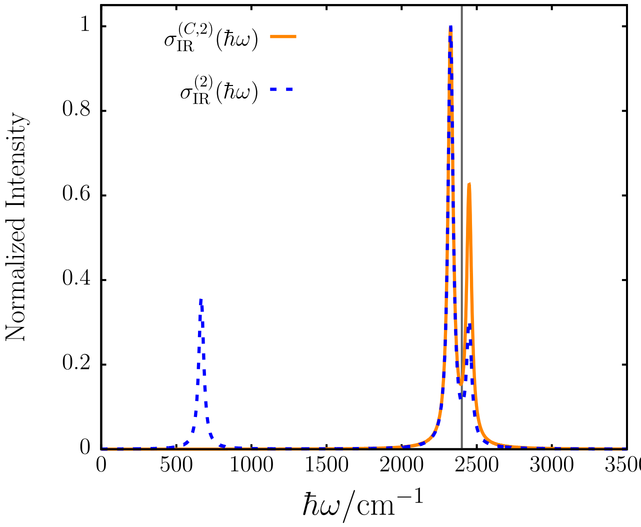

FIG. S1. $\sigma_{\mathrm{IR}}^{(2)}(\hbar\omega)$ and $\sigma_{\mathrm{IR}}^{(C,2)}(\hbar\omega)$ for antisymmetric CO_2-stretching mode under VSC with a single cavity mode, $\hbar\omega_c = \hbar\omega_{\mathrm{as}} = 2400\,\mathrm{cm}^{-1}$, at coupling strength, $g_0 = 0.03\sqrt{E_h}/e\,a_0$. Small peak in $\sigma_{\mathrm{IR}}^{(2)}(\hbar\omega)$ at $\omega = 666\,\mathrm{cm}$ correspond to uncoupled molecular bending mode of CO_2, which is absent in transmission spectrum, $\sigma_{\mathrm{IR}}^{(C,2)}(\hbar\omega)$. The cavity mode frequency is indicated by a grey vertical line.

In Tab.IV, CBO-PT(n) expansion coefficients are given for $n = 1, 2$ at light-matter coupling $g_0 = 0.03\sqrt{E_h}/ea_0$. For CBO-PT(1), lower and upper vibrational polariton states are nearly equally determined by light and matter contributions. This is contrasted by CBO-PT(2) results, where the lower polariton state is dominantly photonic and the upper polariton dominantly molecular in character in line with non-perturbative CBO results[3]. In Fig.S1, $\sigma_{\mathrm{IR}}^{(2)}(\hbar\omega)$ and $\sigma_{\mathrm{IR}}^{(C,2)}(\hbar\omega)$ for antisymmetric CO_2-stretching mode under VSC are shown for the full frequency range, which reveals a small additional peak in $\sigma_{\mathrm{IR}}^{(2)}(\hbar\omega)$ related to bare molecular transitions of uncoupled CO_2 bending modes, which are absent in $\sigma_{\mathrm{IR}}^{(C,2)}(\hbar\omega)$ as they do not carry a photonic component.

TABLE IV. Expansion coefficients of lower (-) and upper (+) vibro-polaritonic states for single CO_2 molecule under VSC with a single cavity mode at $g_0 = 0.03\sqrt{E_h}/ea_0$.

$\lvert a_-^{(1)}\rvert^2$	$\lvert b_-^{(1)}\rvert^2$	$\lvert a_-^{(2)}\rvert^2$	$\lvert b_-^{(2)}\rvert^2$
0.49	0.51	0.38	0.62
$\lvert a_+^{(1)}\rvert^2$	$\lvert b_+^{(1)}\rvert^2$	$\lvert a_+^{(2)}\rvert^2$	$\lvert b_+^{(2)}\rvert^2$
0.51	0.49	0.62	0.38

S5. GENERALIZATION TO MOLECULAR ENSEMBLES

We generalize both CBO-PT(2) Hessians and IR intensities to ensembles of M aligned, *identical* molecules. For M molecules, we have MN_{vib} molecular normal modes, which is the new molecular dimension. Specifically, we can simply understand the ensemble like a large molecule with distinct but degenerate sets of normal modes, such that both normal mode and light-matter interaction block entries, QQ and QC, remain in CBO-PT(1) as derived above. Further, for CBO-PT(2) we consider the ensemble correction

$$E_0^{(2,M)} = \sum_a^M \sum_{\mu_a \neq 0} \frac{|W_{\mu_a 0_a}|^2}{\Delta_{\mu_a}^{(e)}} \quad , \tag{S5.1}$$

where we introduced a sum with index, a, running over all molecules in the ensemble. Now, the single-molecule matrix element in Eq.(S1.43) simply generalizes to

$$W_{\mu_a 0_a} \approx g_0 \sum_{\lambda,k}^{2N_c} \omega_k\, d_{\lambda k}^{\mu_a}\, x_{\lambda k} + \frac{g_0^2}{2} \sum_{\lambda,k}^{2N_c} d_{\lambda k}^{\mu_a} \sum_{i_a}^{N_{\text{vib}}} d_{\lambda k}^{(i_a)}\, Q_{i_a} \quad , \tag{S5.2}$$

and we obtain the same light-matter interaction and molecular corrections, *i.e.*, QQ and QC, as in the single-molecule limit, since differentiation with respect to ∂Q_{i_a} will remove $\sum_a^M$ in both cases. However, we have to account for the ensemble in the cavity contribution, *i.e.*, the first term of the squared matrix element, $W_{\mu_a 0_a}$, which can be now written as

$$\left(\underline{\underline{H}}_{CC}^{(2)}\right)_{\lambda k, \lambda' k'} = -g_0^2\, \omega_k\, \omega_{k'} \sum_a^M \alpha_{\lambda\lambda'}^a \quad , \tag{S5.3}$$

$$= -g_0^2\, M\, \omega_k\, \omega_{k'}\, \alpha_{\lambda\lambda'}^0 \quad . \tag{S5.4}$$

The second line follows from the assumption of identical molecules in the ensemble. Thus, for a single-cavity mode model, the respective *dressed* frequency is then given to leading order in g_0 by

$$\tilde{\omega}_c = \sqrt{\omega_c^2 - g_0^2\, M\, \omega_c^2\, \alpha_{\lambda\lambda}^0} \quad , \tag{S5.5}$$

$$\approx \omega_c \left(1 - \frac{g_0^2}{2} M \alpha_{\lambda\lambda}^0\right) \quad , \tag{S5.6}$$

in close analogy to the main text up to a factor M. In line with the Hessian cavity block, we also have to account for ensemble effects in the cavity intensity. The ensemble cavity component of the generalized dipole moment reads here

$$\underline{D}_{00}^{(C,2)}(\underline{x}) = -g_0 \sum_{\lambda,k}^{2N_c} \omega_k \sum_a^M \alpha_{\kappa\lambda}^a\, x_{\lambda k} \quad , \tag{S5.7}$$

$$= -g_0\, M \sum_{\lambda,k}^{2N_c} \omega_k\, \alpha_{\kappa\lambda}^0\, x_{\lambda k} \quad , \tag{S5.8}$$

where we employ the same identical-molecule argument as for the Hessian correction. Then the related derivative is simply given by

$$\frac{\partial D_{00,\kappa}^{(C,2)}}{\partial x_{\lambda k}} = -g_0\, \omega_k\, M\, \alpha_{\kappa\lambda}^0 \quad , \tag{S5.9}$$

i.e., the cavity intensity depends herein on the ensemble size due to its relation to static polarizabilities.

[1] Fischer, E.W.; Saalfrank, P. Beyond Cavity Born-Oppenheimer: On Nonadiabatic Coupling and Effective Ground State Hamiltonians in Vibro-Polaritonic Chemistry. *J. Chem. Theory Comput.* (2023) 19, 20, 7215-7229.

[2] Haugland, T. S.; Philbin, J. P.; Ghosh, T. K.; Chen, M.; Koch, H.; Narang, P. Understanding the polaritonic ground state in cavity quantum electrodynamics. arXiv preprint arXiv:2307.14822 (2023).

[3] Bonini, J.; Flick, J. Ab initio linear-response approach to vibro-polaritons in the cavity Born-Oppenheimer approximation. *J. Chem. Theory Comput.* **18**, 2764 (2022).

[4] Gaussian 16, Revision C.01, M. J. Frisch, G. W. Trucks, H. B. Schlegel, G. E. Scuseria, M. A. Robb, J. R. Cheeseman, G. Scalmani, V. Barone, G. A. Petersson, H. Nakatsuji, X. Li, M. Caricato, A. V. Marenich, J. Bloino, B. G. Janesko, R. Gomperts, B. Mennucci, H. P. Hratchian, J. V. Ortiz, A. F. Izmaylov, J. L. Sonnenberg, D. Williams-Young, F. Ding, F. Lipparini, F. Egidi, J. Goings, B. Peng, A. Petrone, T. Henderson, D. Ranasinghe, V. G. Zakrzewski, J. Gao, N. Rega, G. Zheng, W. Liang, M. Hada, M. Ehara, K. Toyota, R. Fukuda, J. Hasegawa, M. Ishida, T. Nakajima, Y. Honda, O. Kitao, H. Nakai, T. Vreven, K. Throssell, J. A. Montgomery, Jr., J. E. Peralta, F. Ogliaro, M. J. Bearpark, J. J. Heyd, E. N. Brothers, K. N. Kudin, V. N. Staroverov, T. A. Keith, R. Kobayashi, J. Normand, K. Raghavachari, A. P. Rendell, J. C. Burant, S. S. Iyengar, J. Tomasi, M. Cossi, J. M. Millam, M. Klene, C. Adamo, R. Cammi, J. W. Ochterski, R. L. Martin, K. Morokuma, O. Farkas, J. B. Foresman, and D. J. Fox, Gaussian, Inc., Wallingford CT, 2016.
